# Investigating the Underlying Mechanisms of *Ardisia japonica* Extract’s Anti-Blood-Stasis Effect via Metabolomics and Network Pharmacology

**DOI:** 10.3390/molecules28217301

**Published:** 2023-10-27

**Authors:** Cuiwei He, Erwei Hao, Chengzhi Du, Wei Wei, Xiaodong Wang, Tongxiang Liu, Jiagang Deng

**Affiliations:** 1 School of Pharmacy, Minzu University of China, Beijing 100081, China; 2 Faculty of Pharmacy, Guangxi University of Chinese Medicine, Nanning 530200, China; 3 Guangxi Key Laboratory of Efficacy Study on Chinese Materia Medica, Guangxi University of Chinese Medicine, Nanning 530200, China

**Keywords:** *Ardisia japonica*, blood stasis, non-targeted metabolomics, network pharmacology, inflammation

## Abstract

Objective: Our study aims to assess *Ardisia japonica* (AJ)’s anti-blood-stasis effect and its underlying action mechanisms. Methods: The primary components of AJ were determined using liquid chromatography–mass spectrometry (LC-MS). The blood stasis model was used to investigate the anti-blood-stasis effect of AJ extract. The underlying mechanisms of AJ against blood stasis were investigated via network pharmacology, molecular docking, and plasma non-targeted metabolomics. Results: In total, 94 compounds were identified from an aqueous extract of AJ, including terpenoids, phenylpropanoids, alkaloids, and fatty acyl compounds. In rats with blood stasis, AJ reduced the area of stasis, decreased the inflammatory reaction in the liver and lungs of rats, lowered the plasma viscosity, increased the index of erythrocyte deformability, and decreased the index of erythrocyte aggregation, suggesting that AJ has an anti-blood-stasis effect. Different metabolites were identified via plasma untargeted metabolomics, and it was found that AJ exerts its anti-blood-stasis effect by reducing inflammatory responses through the cysteine and methionine metabolism, linolenic acid metabolism, and sphingolipid metabolism. For the effect of AJ on blood stasis syndrome, the main active ingredients predicted via network pharmacology include sinensetin, galanin, isorhamnetin, kaempferol, wogonin, quercetin, and bergenin, and their targets were TP53, HSP90AA1, VEGFA, AKT1, EGFR, and PIK3CA that were mainly enriched in the PI3K/AKT and MAPK signaling pathways, which modulate the inflammatory response. Molecular docking was also performed, and the binding energies of these seven compounds to six proteins were less than −5, indicating that the chemical components bind to the target proteins. Conclusions: This study suggests AJ effectively prevents blood stasis by reducing inflammation.

## 1. Introduction

In traditional Chinese medicine (TCM), *Ardisia japonica* (AJ) refers to the whole herb of *Ardisia japonica* (Thunb) Blume. The Chinese Pharmacopoeia lists the benefits of AJ, including its ability to dissolve phlegm and relieve coughing, eliminate body heat and humidity, activate blood circulation, and treat blood stasis [1]. AJ is also a commonly used herb among the Zhuang population, which is outlined in monographs and standards such as Gui Materia Medica [2], Selected zhuang Medicines [3], and Quality Standards of Strong Medicines of Guangxi Zhuang Autonomous Region [4]. According to the book “Herb and Tree Prescriptions” [5], AJ has long been applied for treating rheumatism, irreversible paralysis, and chronic coughs [6].

The Zhuang people apply AJ to treat respiratory conditions such as chronic bronchitis and hemoptysis. Several exclusive Chinese medications on the market contain AJ as the primary herb, including compound dwarf earth tea tablets, anti-tuberculosis pills, and bronchitis tablets [7,8]. Several pharmacological properties of AJ, including its antitussive, anti-inflammatory, antioxidant, and hepatoprotective properties, have been demonstrated in animal studies [9,10,11]. AJ mostly affects the liver and lungs. The lung is the respiratory system’s main organ and a repository for various hematopoietic progenitor cells, producing more than half of an animal’s platelets [12]. As a result, pulmonary blood flow and respiratory patency interact with one another [13]. Studies have demonstrated that microvascular thrombosis in patients with coronavirus disease 2019 (COVID-19) plays a crucial role in determining how the disease progresses, and that herbs with cough-relieving, phlegm-reducing, and blood-stasis-resistant effects play an important therapeutic role [14,15]. So, investigating the anti-blood-stasis effect of AJ is important.

Studies have demonstrated that modulating inflammatory responses and enhancing blood rheology were two underlying resistance mechanisms against blood stasis [16,17,18]. The chemical composition of AJ includes coumarins, triterpenoids, quinones, flavonoids, volatile oils, and other constituents, which have been shown to ameliorate coughing, clear phlegm, protect the liver, and serve as anti-inflammatory agents.

Metabolomics can be used to understand endogenous differential metabolite profiles in biological systems. The many-faceted effects of herbal medicines can be consistently studied through metabolomics’ comprehensive viewpoint. Metabolomics presents a fresh and promising approach to assessing herbal medicines’ therapeutic benefits and elucidating their underlying mechanisms.

The application of metabolomics methods to investigate AJ’s blood-activating and stasis-relieving properties has yet to be reported. We examined the anti-blood-stasis mechanism of AJ in this work and demonstrated that flavonoids in AJ are its primary active component.

## 2. Results

### 2.1. Main Components in AJ

The AJ water extract was analyzed using UHPLC-Q extractive/MS, and base peak ion (BPI) chromatograms were obtained in both positive and negative ion modes. A total of 94 compounds were identified in AJ (Table 1) through database comparison.

### 2.2. Anti-Blood-Stasis Effect of AJ

**Figure 1:**
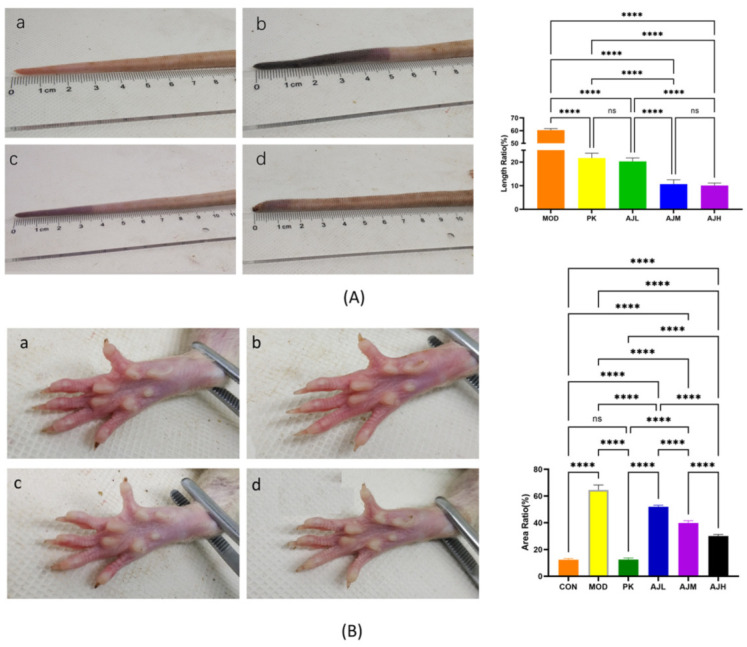
AJ decreased blood-stasis in rat tail and paw. (**A**) Analysis of blood-stasis in rat tail. (**B**) Analysis of blood-stasis in rat paw (a: CON group, b: MOD group, c: PK group, d: AJH group). Data are presented as mean ± standard deviation (SD) (*n* = 10). **** *p* < 0.0001, control by one-way ANOVA.

After prophylactic administration and modeling of the rats, the rats in the CON group had a good mental status and normal hair color, while the rats in the MOD group displayed huddling movements, redness of the extremities, and obvious thrombi on the tail. All groups revealed varying degrees of stasis within the epidermis and cyanosis of the epidermis except for the CON group, which had a reddish paw color and no stasis. The AJL, AJM, and AJH groups significantly ameliorated ratios A and L (*p* < 0.001). The blood stasis symptoms were ameliorated significantly compared to those in the MOD group. The results are presented in Figure 1.

### 2.3. Pathological Section

**Figure 2:**
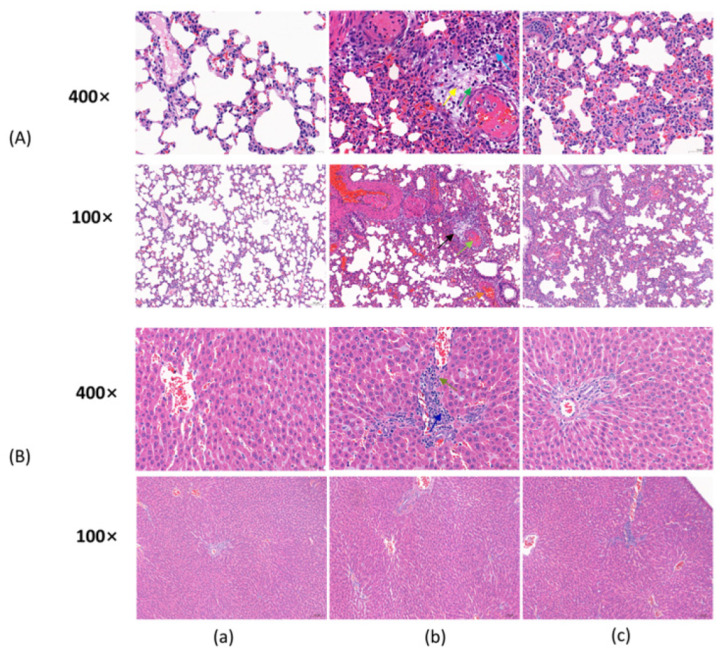
AJ reduced inflammation in the lungs and liver of blood stasis rats. (**A**) Pathologic section of lungs (enlargement 400×, top row; enlargement 100×, bottom row). (**B**) Pathologic section of live (enlargement 400×, top row; enlargement 100×, bottom row). (a) CON group. (b) MOD group. (c) AJH group. In (**A**) top row (b): lymphocytes (↑), neutrophils (↑), fibrous tissue degeneration and necrosis of the vascular epicardium (↑); in (A) bottom row (b): infarction (↑), vascular congestion (↑), arteriosclerosis (↑); in (**B**) top row (b): lymphocyte (↑), neutrophil (↑).

Lungs: The CON group had normal alveolar septa, no cavity exudate, no congestion and inflammatory infiltration in the pulmonary interstitium, and few aggregated erythrocytes in the small pulmonary veins. Thrombosis and blood vessel obstruction were observed in the MOD rats, as well as vascular congestion and erythrocyte accumulation. A few of the extravascular sides had loose connective tissue necrosis, karyolytic fragmentation, edema, widened intercellular spaces, and inflammatory cell accumulation in the lumen. Rats in the AJH group had substantially less inflammatory cell infiltration, and the alveolar structure was intact, as shown in Figure 2A.

Liver: The CON group rats had normal hepatic lobules and a hepatocyte structure. In the MOD group, inflammatory cell infiltration, which included mononuclear, round, deeply stained lymphocytes and rod-shaped, nuclear neutrophils, was observed around the portal area. A clear improvement in inflammation was observed in the rats of the AJH groups, as shown in Figure 2B.

### 2.4. Hemorheological Index

#### 2.4.1. Effect on Blood Viscosity

**Figure 3:**
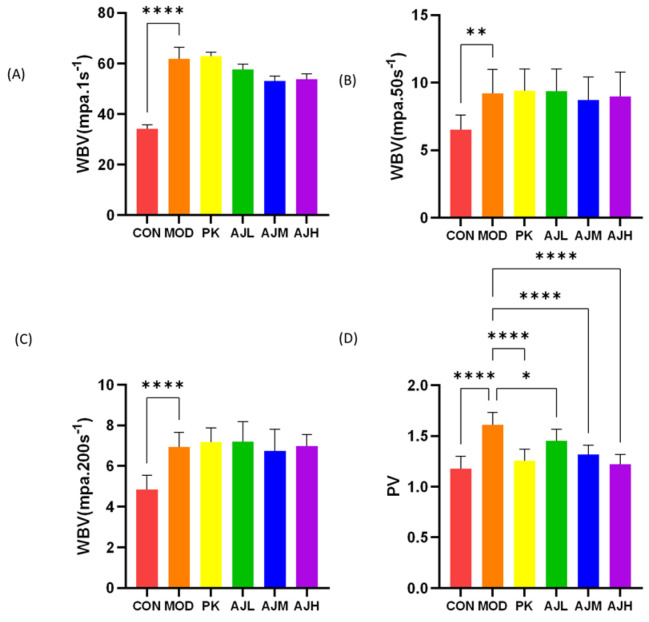
AJ reduced plasma viscosity in blood stasis rats. (**A**) WBV (mpa·1 s^−1^). (**B**) WBV (mpa·50 s^−1^). (**C**) WBV (mpa·200 s^−1^). (**A**–**C**) There is significant difference (*p* < 0.0001) in WBV between CON group and MOD group, suggesting successful modeling. (**D**) PV of the AJM and AJH groups was significantly reduced vs. MOD group (*p* < 0.0001), indicating that AJ can reduce plasma viscosity. The results are presented as mean ± standard deviation (SD) (*n* = 10). * *p* < 0.05, ** *p* < 0.01, **** *p* < 0.0001, control by one-way ANOVA.

In Figure 3, there was no significant difference in WBV (*p* > 0.05) between the MOD group and the AJL, AJM, and AJH groups, indicating that AJ has no significant improvement effect on the whole blood viscosity in rats with blood stasis. The PV (*p* < 0.001) of the AJM and AJH groups was significantly reduced compared to that of the MOD group, indicating that AJ can reduce plasma viscosity and improve in the condition of rats with blood stasis.

#### 2.4.2. The Impact on Red Blood Cell Aggregation and Deformability Indicators

**Figure 4:**
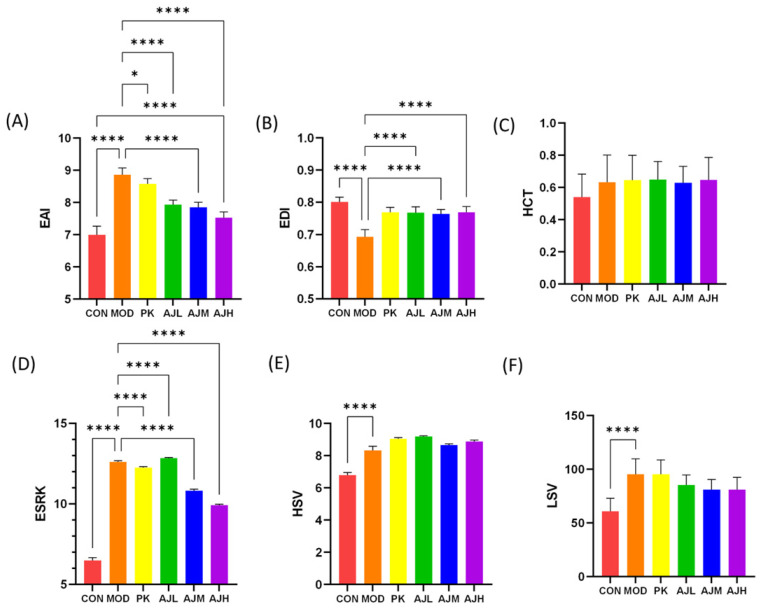
The effect of AJ on the hemorheological index in rats. (**A**) EAI reduced significantly (AJH group vs. MOD group). (**B**) EDI rose remarkably (AJL, AJM and AJH groups vs. MOD group). (**C**) HCT. (**D**) ESRK reduced significantly (AJL, AJM and AJH groups vs. MOD group, *p* < 0.0001). (**E**) HSV. (**F**) LSV. The results are presented as mean ± standard deviation (SD) (*n* = 10), * *p* < 0.05, **** *p* < 0.0001, control by one-way ANOVA.

The red blood cell aggregation index (EAI) was substantially lower in the AJH group compared to that in the MOD group (*p* < 0.001), and the red blood cell deformation index (EDI) was substantially higher in the AJL, AJM, and AJH groups compared to that in the MOD group (*p* < 0.001), indicating that the AJH group could significantly enhance red blood cell deformability, decrease red blood cell aggregation, and prevent embolization. The decrease in the erythrocyte sedimentation rate (ESRK) in the MOD group (*p* < 0.0001) indicates an improvement in the acute inflammation in rats, as shown in Figure 4.

### 2.5. Non-Targeted Metabolomics

#### 2.5.1. Stability of Equipment

Technical replicates, metabolite extraction, and detection repeatability were assessed by performing overlap display analysis using the total ion chromatograms (TICs) of the various quality control (QC) samples obtained through the mass spectrometric detection assay. The good overlap of the TIC plots of various samples is presented in Figure S1 of the Supplementary Materials, and the instrument’s high stability ensured the data’s repeatability and reliability.

#### 2.5.2. Principal Component Analysis

**Figure 5:**
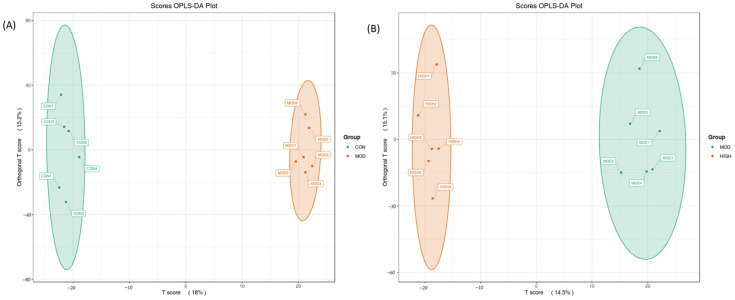
Data distribution in OPLS-DA model. (**A**) CON group vs. MOD group; (**B**) MOD group vs. AJH group. Metabolite samples from various groups were distinguished while samples from the same group gathered together.

The concentration of the metabolite distribution within groups and differences between groups was examined using OPLS-DA analysis on the samples (including the quality control samples). Figure 5 demonstrates that samples from various groups could be distinguished while samples from the same group had a more concentrated distribution of metabolites, suggesting that the collected data may be utilized to identify markers that differ between groups.

#### 2.5.3. Screening and Identification of Potential Biomarkers

The OPALS-DA method was applied to examine group differences and determine how AJ affects the rat endogenous drug metabolism. Potential metabolites significantly contributing to clustering and differentiation were chosen using their variable importance prediction (VIP) values and *p*-values. The metabolites with a VIP > 1 and *p* < 0.05 were potentially discriminating (Table 2).

**Figure 6:**
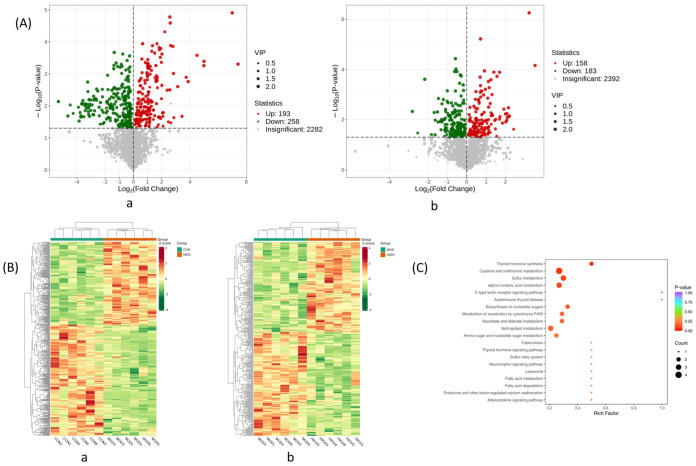
Analysis of differential metabolites in non-targeted metabolomics. (**A**) Volcano map of differential metabolites. (a) CON group vs. MOD group. (b) MOD group vs. AJH group. (**B**) Thermal images of differential metabolites. (a) CON group vs. MOD group. (b) MOD group vs. AJH group. (**C**) KEGG analysis of metabolic pathways.

In Figure 6A, the distinct metabolite expression trends between the control and model groups, together with those of the model and dose groups, were shown on volcano plots, and each dot stands for a particular metabolite, on the left side of the vertical coordinate are the metabolites that were downregulated, and on the right are those that were upregulated. The differential metabolite-related heat images are presented in Figure 6B to show the relative abundance of possible biomarkers in each sample based on the metabolites’ relative intensities in the normal, model, and dosed groups. In total, 50 differentiation metabolites were mapped to their biological pathways to gain a better understanding of the biochemical reactions triggered by AJ based on KEGG analysis. The size of the bubbles in the figure represents the number of genes enriched in this pathway, and the color of the bubbles represents significance. The redder the bubbles, the smaller the *p*-value and the more significant the statistical difference, as shown in Figure 6C.

### 2.6. Network Pharmacological Analysis

**Figure 7:**
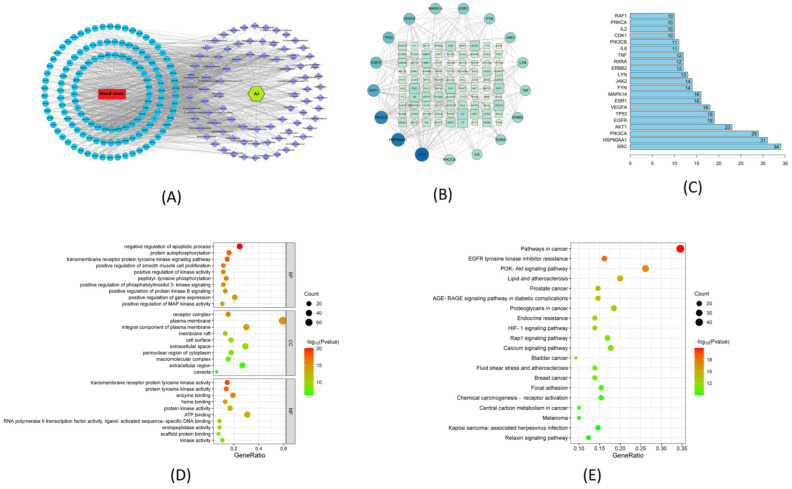
Network pharmacological analysis of AJ’s potential downstream targets. (**A**) “Drug-Component-Target-Disease” network. (**B**) PPI diagram summarizing the intersection of mall molecules and proteins associated with blood stasis and AJ. (**C**) Core targets. (**D**) Enrichment of Gene Ontology (GO) categories among potential downstream targets of AJ that have been associated with blood stasis. Bubble plot of the top 10 GO categories in each of the three domains of biological processes (BP), cellular components (CC), and molecular functions (MF). (**E**) KEGG signaling pathways analysis of AJ’s anti-blood-stasis.

The LC-MS research yielded 94 chemical compositions from AJ via the anti-blood-stasis Target-Component-Herb and PPI network analysis. After deleting duplicate genes, we standardized 685 AJ targets to recognize gene symbols using the SwissTargetPrediction database (http://www.swisstargetprediction.ch, accessed on 10 December 2022). After duplicate genes were removed, 659 possible blood stasis targets were located using the Gene Cards, OMIM, and DisGeNET databases. The Venn charts revealed an intersection of 130 genes between AJ’s active targets and diseases associated with blood stasis. Regarding anti-blood-stasis effectiveness, 72 components and 130 targets of AJ were present in the Target-Component-Herb network. Sinensetin, galangin, isorhamnetin, kaempferol, morin, wogonin, quercetin, and bergenin were the top eight ingredients. The “Drug-Component-Target-Disease” network is shown in Figure 7A. In the PPI network, a target symbolizes each node, and the nodes’ edges express the connections between targets. The degree of each node indicates how many links it has. The likelihood that a node in the graph would be a core target is increased alongside node size and degree values. Figure 7B shows the total number of nodes and bar edges for the core protein targets, and the Figure 7C shows the core targets, such as SRC, HSP90AA1, PIK3CA, EGFR, TNF, MAPK14, MMP2, IL-6, and IL-2. According to the GO results, the top five items related to biological processes (BP) are the control of inflammatory responses, lipopolysaccharide reaction, external stimulus response, hormone response, and cellular lipid response. Membrane rafts, vesicle lumen, serine-type peptidase complexes, and the extracellular matrix were the main areas of CCS enrichment (Figure 7D). The top 20 components of the KEGG pathway enrichment analysis were chosen based on the logo value to create a bubble diagram, showing that the AJ component action targets are involved in cancer pathways, EGFR tyrosine kinase inhibitor resistance, PI3K-Akt signaling, lipids and atherosclerosis, prostate cancer, and the AGE-RAGE signaling pathway in the diabetic complication pathways. The size of the bubbles in the figure represents the number of genes enriched in this pathway, and the color of the bubbles represents significance. The redder the bubbles, the smaller the *p*-value and the more significant the statistical difference (Figure 7E).

### 2.7. Molecular Docking Results

**Figure 8:**
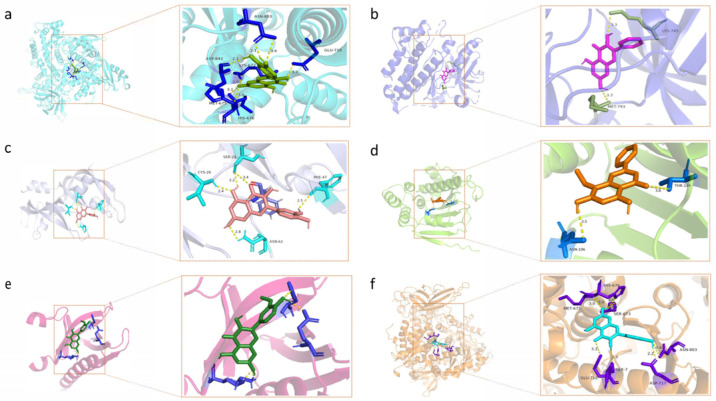
Visual analysis of molecular docking. (**a**) PIK3CA-quercetin; (**b**) EGFR-galangin; (**c**) VEGFA-quercetin; (**d**) HSP90AA1-quercetin; (**e**) AKT1-isorhamnetin; (**f**) PIK3CA-kaempferol.

**Figure 9:**
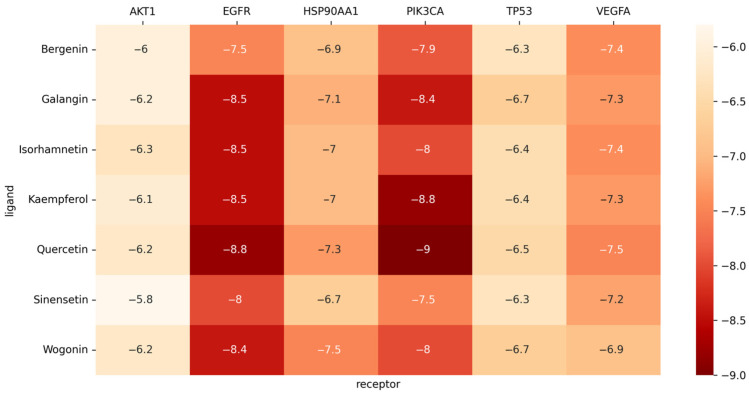
The binding energy between the substances and the targets are less than −5.0, indicating the binding was stable.

The human-originated protein molecules TP53, HSP90AA1, VEGFA, AKT1, EGFR, and PIK3CA were found in the PDB. Using PyMOL software 2.5, superfluous chains on protein and ligand molecules were removed, and water was dehydrogenated. Six protein and seven drug molecules (sinensetin, galangin, isorhamnetin, kaempferol, wogonin, quercetin, and bergenin) were then imported, docking boxes were built, and molecular docking was carried out using AutoDock Vina. As depicted in the image, all the binding energies were less than −5.0, which meant the binding of the ligand to the receptor was stable (Figure 8 and Figure 9).

## 3. Materials and Methods

### 3.1. Herbal Remedies and Techniques of Preparation

AJ aqueous extract: The dry medicinal powder of AJ (1.7 kg) was extracted twice with water via refluxing (1 h each). The solvent was then removed with a water bath and concentrated into an infusion, and the hot extract was concentrated via evaporation in a water bath. The crude extract was formulated as an AJ aqueous extract (containing the crude drug amount of 0.8 g/mL).

Peach kernel seed aqueous extract: The peach kernel seed aqueous extract was made similarly (containing a crude drug amount of 0.8 g/mL).

The 1% carrageenan suspension: A total of 1 g of carrageenan was added to 100 mL of water and stirred ultrasonically at 25 °C for 10 min to form a 1% suspension.

The 20% aqueous yeast slurry: A total of 20 g of yeast was added to 100 mL of water and stirred ultrasonically at 25 °C for 10 min to form a 20% suspension.

### 3.2. Experiments on Animals

#### 3.2.1. Animals

From Guangxi Medical University, 60 male Sprague Dawley (SD) rats weighing 200 g were obtained (approval number: SCXKgui2020-0003). All rats lived in a room with ambient humidity of 40–50%, a temperature of 20–25 °C, and a 10–14 h light-to-dark cycle. Food and water were freely available to the rats. The Ethics Committee of the Guangxi University of Traditional Chinese Medicine examined and approved all animal experimentation methods.

#### 3.2.2. Animal Care and Diet

A total of 60 SD rats were randomly assigned to 6 groups: the control (CON), model (MOD), peach kernel positive control (PK), AJ low dose (AJL), AJ medium dose (AJM), and AJ high dose (AJH) groups. The rats in the AJ groups were given the AJ aqueous extract (AJL 31.25 g/kg, AJM 62.50 g/kg, and AJH 93.75 g/kg), the PK group was given the peach kernel seed aqueous extract (9.375 g/kg), and the CON group and MOD group were given the corresponding blank solvent (distilled water). All rats were gavaged at 20 mL/kg 2 times/D for 7 d. From the 5th to 7th d of administration, 1% carrageenan suspension was injected into the cavity of the rats intraperitoneally with 5 mL/kg 1 time/D in all groups except for the CON group; then, they received 10 mL/kg of a 20% aqueous slurry of yeast subcutaneously in the back 24 h after the 7th d gavaged of AJ aqueous extract and injection of the carrageenan suspension, and an index observation for each group was performed 6 h later.

#### 3.2.3. Rat Appearance Index Detection

(1) Observation of Rat State. After 7 d of modeling, the rats’ mental status, activity, and signs were observed in a quiet environment. Images of the rats’ whole body, hair, paws, tail, and other parts were taken with a digital camera at fixed angles and under light after the rats were anesthetized and placed in a cold-light-source photographing box. The images were processed and analyzed using a self-developed “Rat signs collection and analysis system (Chongqing Cente Technology Co. Ltd., Chongqing, China)”. The length of the rat tail stasis and the area of the rat paw stasis were measured, and the ratios of blood stasis length to whole tail length (L) and blood stasis area to full claw area (A) were calculated and compared among the groups.

The ratio L = blood stasis length/whole tail length.

The ratio A = blood stasis area/full claw area.

(2) Pathological section. Rat liver and lung tissues were removed 6 h after the dry yeast injection and fixed in a 4% paraformaldehyde solution. The liver and lung tissues above were prepared as paraffin sections, and hematoxylin–eosin (HE) staining was used to observe the samples under a light microscope.

(3) Detection of hemorheological indexes. The rats were anesthetized by intraperitoneal injection. Blood was collected from the abdominal aorta into 5 mL vacuum heparin anticoagulant tubes, which were centrifuged for 10 min at 3000 rpm for the corresponding detection of indexes including whole blood viscosity (WBV), plasma viscosity (PV), low shear viscosity (LSV), hematocrit RBC (HCT), high shear viscosity (HSV), the erythrocyte aggregation index (EAI), the erythrocyte deformation index (EDI), and the k-value of the erythrocyte sedimentation equation (KESR).

#### 3.2.4. Metabolomics Blood Sampling

Blood was collected from the abdominal aorta into 5 mL vacuum heparin anticoagulant tubes, which were centrifuged for 10 min at 3000 rpm, and then the plasma was immediately frozen in liquid nitrogen and kept at −80 °C in a refrigerator.

### 3.3. Analysis of AJ Extract Components

Chromatographic separation (1290 UHPLC, Agilent, Santa Clara, CA, USA) was carried out on the Acquity UPLC BEH C18 (2.1 × 100 mm, 1.7 μm, Waters, Milford, MA, USA) with the following conditions: 0.5 mL/min flow rate, column temperature of 30 °C, a mobile phase of water (A) and acetonitrile (B) (phase A and B both contain a 0.2% solution of formic acid), and a gradient elution of 85–25% for 0–11 min (A), 25–2% for 11–12 min (A), and 2–85% for 12–16 min (A).

Mass spectrometry was executed using mass spectrometric detection and analysis in both positive and negative ionization mode using a Q Exactive Focus detector with an NCE source (Thermo Fisher Scientific, Waltham, MA, USA). The operating parameters were as follows: complete scan mode with a scan range of *m*/*z* 100–1500 Da, capillary temperature of 350 °C, scanning voltage of 4 kV in negative/positive ion mode, sheath gas of 45 ARB, resolution of 7 × 10^4^, and analysis cycle of 16 min.

### 3.4. Analysis of Non-Targeted Metabolomics

The non-targeted metabolomics analysis used plasma from the CON, MOD, and AJH groups. The −80°C freezer’s plasma test samples were defrosted on ice and then reheated to room temperature (all subsequent manipulations were performed on ice). After vortexing for 10 s, 50 μL samples were pipetted to another centrifuge tube, which were then spiked with 300 mL of an internal standard extraction solution of 20% acetonitrile and methanol. Next, 200 μL of the supernatant was pipetted into the other tube after being vortex mixed for 3 min, centrifuged at 12,000 t/min for 10 min at 4 °C, left in the freezer at −20 ° C for 30 min, and recentrifuged at 12,000 rpm for 3 min at 4 °C. Afterward, 180 μL of the supernatant was pipetted into the lining of the matching injector vial for analysis.

The metabolomic profiling was performed on a UHPLC-Q-Exactive MS system (Thermo Fisher Scientific). The samples were separated with a UPLC BEH C18 column (1.7 μm, 2.1 × 100 mm, Waters, USA). The flow rate was 0.40 mL/min, and the injection volume was 2 μL. Column temperature: 40 °C. Sample gradient elution in the mobile phase: 0.2% formic acid and water (A), and 0.1% formic acid and acetonitrile (B). Elution conditions: 0–11 min, 99–10%, A; 11–12 min, 10%, A; and 12–14 min, 10–95%, A. After separation, the samples were examined utilizing the positive and negative ion modes of an ESI mass spectrometer, the Q-Exactive from Thermo Fisher Scientific. Proteowizard was used to convert the raw mass spectrometer data into the mzXML format, and peak extraction, alignment, and retention time correction were performed using the XCMS program. Peak regions in each sample set were filtered for peaks with missing values higher than 50% and adjusted using the “SVR” approach. After correcting the filtered peaks, information on metabolite identification was retrieved via lab self-building databases, public libraries, AI prediction libraries, and the metDNA technique. The supernatant of each test sample was resuspended in 10 μL and used as a quality control (QC) sample. One QC sample was introduced every 15 samples to assess the stability of the equipment.

### 3.5. Network Pharmacology and Molecular Docking

#### 3.5.1. Prediction of AJ’s Drug Targets

Using “blood stasis” and “thrombus” as keywords, potential targets related to blood stasis were collated from the Gene Cards database (correlation score > 1), OMIM database, and DisGeNET database; then, duplicate entries were removed, and a disease target gene dataset was established. The AJ components and disease-related targets were intersected to obtain common target candidates. Targets with combined scores greater than the mean of 0.900 and degree values more than the mean were chosen as key targets after analyzing the “degree” and “Formation score” values. To show the connections between the main protein targets of the drug, protein–protein interaction (PPI) networks were created using STRING (https://string-db.org (accessed on 10 January 2023)) and displayed using Cytoscape (Cytoscape Consortium, San Diego, CA, USA). Moreover, compound-target-pathway networks were also created using Cytoscape. Compounds and proteins were represented in the graph network as nodes, while interactions between compounds and proteins were shown as edges. Nodes in the graphical networks stand in for the many substances, targets, and associated disorders. The edges between them show the linkages between the compound target and target diseases. Version 3.2 of the KEGGscape plugin for Cytoscape was used to map the relevant disease pathway.

#### 3.5.2. GO and KEGG Analysis

Gene ontology (GO) analysis was performed on the common target candidates of the AJ components and blood stasis diseases using the metascape database. The probable target genes’ involvement in the biological process (bioprocess BP), cellular component (CC), and molecular function (MF) was examined using GO analysis. Using an online bioinformatics program (http://www.bioinformatics.com.cn/, (accessed on 12 January 2023)), the ascending *p*-values were ordered using *p* < 0.01 as the border, and the top 20 most significant pathways were made visible in a bubble chart. The bubbles’ color and size represented the *p*-value and the number of targets enriched in the chosen pathway.

#### 3.5.3. Molecular Docking

Six core proteins identified as target proteins in the PPI network were docked to confirm the affinity between the AJ components and illness cross-targets. The first seven core compounds were chosen as therapeutic small molecules. To find the 3D structural formula of the proteins, the protein structure database (PDB; https://www.rcsb.org/, (accessed on 22 January 2023)) was searched. PyMOL software was then used to remove redundant chains, delete ligands, dehydrate and hydrogenate the protein molecules and drug molecules, and set the docking box, and AutoDock Vina (https://vina.scripps.edu/, (accessed on 22 January 2023)) was used to identify molecules for semi-flexible docking to find the binding energy and position of the AJ component.

### 3.6. Statistical Analysis

The data were checked for normality and homogeneity of variance using SPSS 25.0 software (SPSS Inc., Chicago, IL, USA). Before using one-way ANOVA to analyze the remaining data, variance (ANOVA) analysis was utilized to evaluate the data for repeated measurements. Comparisons were conducted using the least significant difference approach. Item-by-item statistical analysis was performed using non-parametric tests for independent samples if the data lacked variation or were not normally distributed. Plotting was carried out using the GraphPad Prism 9.0 (San Diego, CA, USA) program.

## 4. Discussion

Our study investigates the potential mechanisms of AJ in combating blood stasis through animal experiments, plasma metabolomics, network pharmacology, and molecular docking.

In animal experiments, after intragastric administration of AJ to blood stasis rats, there was a considerable increase in the erythrocyte deformation index (EDI), a significant decrease in the erythrocyte aggregation index (EAI), the plasma viscosity (PV) was reduced, and the congestion area in tail and paw bleeding was reduced, indicating that AJ has the effect of promoting blood circulation and resolving blood stasis. Meanwhile, the erythrocyte sedimentation rate (ESRK) of rats decreased, and the pathological section analysis results showed that the inflammatory reactions in the liver and lungs of blood stasis rats were reduced. Therefore, we believe that AJ may exert its anti-blood-stasis effect through anti-inflammatory effects.

In plasma metabolomics, different metabolites from the AJ and MOD groups were detected and enriched on 20 KEGG pathways, including thyroid hormone synthesis and the adipocytokine signaling pathway.

Multiple pathways suggest that the efficacy of AJ encompasses multiple aspects such as affecting protein synthesis, causing vascular protection, and regulating immune homeostasis, demonstrating the multi-target effect of traditional Chinese medicine. The metabolites with the greatest difference include L-palmitoyl carnitine, quinolinic acid, D-mannose, 7-methylxanthine nucleoside, ceramide, and multiple amino acid. L-palmitoyl carnitine is a byproduct of long-chain acyl carnitine and the fatty acid metabolism, which accumulates in the muscle membrane during ischemia and disrupts the lipid environment of the membrane. L-palmitoyl carnitine can affect the inflammatory response by participating in the fatty acid metabolism. The translocation of long-chain fatty acids into the mitochondrial matrix is dependent on carnitine and involves the translocation process, which is necessary for the synthesis of energy from long-chain fatty acids [19,20]. The interruption of the transportation of long-chain fatty acid energy to the mitochondrial matrix, which in turn affects energy production, may be caused by an increase in plasma L-palmitoyl carnitine levels and a decrease in octadecanoyl carnitine levels in a chronic, unpredictable, mild-stress rat model [21]. The level of L-palmitoyl carnitine in the AJH group showed significant changes, indicating that L-palmitoyl carnitine is important in physiological processes and helps to transfer long-chain fatty acids from the cytoplasm to mitochondria during fatty acid oxidation. Pyruvate metabolism produces 2,8-hydroxyquinoline, which is then decomposed and metabolized into quinolinic acid. Quinolinic acid has catabolic, antibacterial, anti-tumor, antifungal, and anticancer effects, as well as anti-inflammatory properties [22]. Hypoxia is one of the key features of osteoarthritis, and in mouse models of the disease, it hinders the formation of quinolinic acid from 2,8-hydroxyquinoline, exacerbating the inflammatory response [23]. After intragastric administration of AJ, the plasma levels of quinolinic acid in the study rats decreased, indicating that 2,8-hydroxyquinoline inhibited the production of quinolinic acid and reduced the inflammatory response. Many cells use certain carbohydrates as energy sources, including glucose and D-mannose [24]. In the AJH groups we studied, the concentration of carbohydrate-related metabolites (such as D-mannose) was significantly lower than that of the MOD group, as these sugars may be metabolized into lactic acid and malic acid to provide additional energy. Mannose has been proven to be an analgesic drug that can prevent LPS-induced lung injury [25], and D-mannose depletion may help prevent the onset of inflammation [26]. 7-methylxanthine nucleoside has been shown to significantly reduce IL-1 levels in the plasma and blood glucose levels in patients with hyperglycemia [27]. The concentration of 7-methylxanthine nucleoside in the AJH groups is higher than that in the MOD group, which may help reduce inflammatory reactions in blood stasis rats. In addition, we found that after administering the AJ intervention, the plasma ceramide levels in rats were significantly downregulated. Icosahexaenoic acid produces ceramide, which is a key molecule in fatty acid metabolism. Ceramide is an important regulatory lipid in the brain and central nervous system with anti-inflammatory, neuroprotective, and sleep cycle control effects. Several studies have shown that a large amount of ceramide accumulates in the cerebrospinal fluid of sleep-deprived mice [28], and the plasma ceramide levels in Alzheimer’s disease patients are significantly increased [29]. In elderly AD animal models, dietary administration of ceramide can prevent β Amyloid protein (Aβ) production, plaque deposition, and cerebral amyloid angiopathy, which increases the brain blood volume [30], meaning that AJ may alter ceramide levels to affect the inflammatory response. After the AJ intervention, significant changes were observed in the levels of various amino acids such as S-sulfo-L-cysteine, D-cysteine, and 3-iodothyrosine. Amino acids, as basic substrates and regulatory elements, are crucial in various metabolic pathways [31,32]. Therefore, the differential metabolites between the AJ and MOD groups are mainly enriched in metabolic pathways including the cysteine and methionine metabolism, linolenic acid metabolism, and sphingolipid metabolism pathways. AJ may mediate inflammation and participate in the process of the anti-blood-stasis effect through these metabolic pathways.

Based on the network pharmacology analysis, AJ might affect SRC, HSP90AA1, AKT1, EGFR, MAPK14, TNF, IL6, and IL2 in the cell periphery and cell membrane, as it is involved in the PI3K-Akt signaling pathway and MAPK signaling pathway. EGFR (Epidermal Growth Factor Receptor) is a receptor for epithelial growth factor (EGF) cell proliferation and signal transduction [33]. After activation, it transforms from monomers to dimers and undergoes auto-tyrosine phosphorylation by phosphorylating downstream proteins, activating the PI3K-AKT-mTOR signaling pathway, and reacting to inflammation through catalysis [34]. The PI3K-Akt signaling pathway plays an important role in regulating airway inflammation in chronic obstructive pulmonary disease [35]. Src kinase is a member of SFK that controls cell growth through signal transduction. Knocking down Src or dasatinib (an Src inhibitor) inhibits the activation of p38, ERK, and AP-1, as well as the expression of COX-2 [36,37], indicating that Src may play a role in the initiation and progression of inflammation. TNF plays an important role in acute and chronic inflammatory diseases and many autoimmune diseases are associated with changes in TNF expression [38,39]. HSP90AA1 is a member of the companion family, whose function is to guide the late tertiary folding of various proteins. HSP90AA1 can guide the NF-κB folding of conformations of members related to the signaling pathway, such as receptor-interacting proteins and inhibitor of κB kinase (IKK), which can degrade after HSP90AA1 inhibition, blocking NF-κB. The signaling pathway is activated and reduces the production of inflammatory factors via macrophages and other cell types [40,41]. Therefore, increasing evidence suggests that HSP90AA1-targeting agents can be used to treat inflammatory diseases. MAPK14, also known as p38, is an enzyme encoded by the MAPK14 gene and a member of the MAPK kinase family. Studies have shown that MAPK14 is associated with the cellular inflammatory response and apoptosis [42]. When myocardial cells are stimulated by external stimuli, a series of inflammatory reactions occur, and at the same time, the level of MAPK14 is significantly increased. An increase in MAPK14 activity was also observed in animal models of heart failure and myocardial biopsies of heart failure patients. MAPK14 inhibitors can inhibit the inflammatory response and apoptosis of myocardial cells [43,44].

Regarding potential pathways, the PI3K/Akt signaling pathway is widely involved in the inflammatory response process. The activation of the PI3K/Akt signaling pathway requires Akt phosphorylation, and the Akt protein kinase is a downstream effector of PI3K signaling [45,46]. Activated Akt is involved in multiple processes including cell proliferation, apoptosis, migration, and the inflammatory response. Research has shown that signaling pathways, including the key targets PI3K and Akt, are involved in multiple stages of the pulmonary airway inflammation response [47] and can reduce myocardial cell apoptosis [48]. After simulating an ischemia-reperfusion injury, luteolin significantly enhanced the phosphorylation levels of PI3K and AKT in rat embryonic myocardial cells (H9C2), reduced the inflammatory response, and improved the ischemia-reperfusion injury [49]. Meanwhile, the induced activation of PI3K/AKT and as well as the inhibition of NF-κB inhibits cell apoptosis and the expression of inflammatory cytokines, ultimately protecting H9C2 cells from ischemia-reperfusion injury [50]. Other studies have shown that the activation of the PI3K pathway regulates the balance between pro-inflammatory and anti-inflammatory responses after the lipopolysaccharide stimulation of cells [51]. This pathway is also linked to the NF-κB interaction in regulating the inflammatory response [52]. The MAPK signaling system, including downstream TNF, IL-6, iNOS, and COX-2, is an important signaling pathway that regulates the production of several pro-inflammatory cytokines and mediators [53]. A key step in activating MAPK is IKK α for I κ B α downstream phosphorylation. Reducing the I κ B α phosphorylation can significantly reduce NF-κB downstream of the MAPK activation. In addition, ERK, JNK, and p38 (MAPK14) are downstream parts of the MAPK signaling pathway, which regulate the expression of inflammation-related genes, thereby increasing the production of pro-inflammatory cytokines [54,55]. AJ can promote the phosphorylation of MAPK14, ERK, and JNK in cells, resulting in anti-inflammatory effects.

In addition, researchers have studied the relationship between inflammation and blood stasis syndrome and found that an inflammatory response is an important mechanism for drugs to resist blood stasis. A Danhong Injection, a blood activating and stasis resolving drug, can significantly reduce the levels of IL-6, TNF, and IL-8 in the serum of blood stasis rats, as well as inhibit inflammation and platelet aggregation, thereby reducing the immune response and peroxidation, protecting vascular endothelium and organ function, and preventing and treating cardiovascular diseases [15]. Xuefu Zhuyu Tang is used to promote blood circulation and eliminate blood stasis. Experiments have shown that it has anti-inflammatory effects and can reduce neuroinflammation in APP/PS1 transgenic mice [56]. The inflammatory factor intercellular adhesion molecule-1 (ICAM-1) can distinguish between two types of blood stasis syndrome, qi stagnation and blood stasis syndrome (QSBS) and qi deficiency and blood stasis syndrome (QDBS) [57], indicating a close relationship between inflammation and blood stasis. Yu Moet added blood-activating drugs to the clinical treatment of respiratory inflammation in Western medicine, achieving satisfactory results in shortening the course of the disease, alleviating symptoms, promoting inflammation recovery, and avoiding complications [58]. These studies indicate that blood stasis is closely related to inflammation in specific protein components, clinical treatment, and inflammatory response situations.

Meanwhile, when we used molecular docking methods for targeted prediction, we found that chemical substances in AJ, including passionetin, galangin, isorhamnetin, kaempferol, baicalin, quercetin, and bergenin, could bind well to AKT1, EGFR, HSP90AA1, PIK3CA, TP53, and VEGFA proteins, suggesting that flavonoids and coumarin compounds in AJ may be active ingredients for treating blood stasis.

## 5. Conclusions

In summary, this study examined the anti-blood-stasis effect of AJ and investigated the plasma metabolic differences between rats and blood stasis model rats after taking AJ. The mechanism of AJ’s anti-blood-stasis effect was also explored using network pharmacology. AJ alleviated congestion symptoms in blood stasis model rats and improved the inflammatory response through the fatty acid metabolism, amino acid metabolism, PI3K/AKT signaling pathway, and MAPK signaling pathway, exhibiting an anti-blood-stasis effect. Passiflorin, galangin, isorhamnetin, kaempferol, scutellarin, quercetin, and bergenin may be important components in the fight against blood stasis.

## Figures and Tables

**Table 1:** Summary of the compounds of AJ.

No.	R_t_ (min)	Name	Formula	Ms/Ms	*m/z*
1	11.44	Arachidonic acid	C_20_H_32_O_2_	[M − H]^−^	303.2325
2	1.28	Benzoic acid	C_7_H_6_O_2_	[M − H]^−^	121.0296
3	11.19	Isopalmitic acid	C_16_H_32_O_2_	[M − H]^−^	255.2327
4	0.73	Kynurenic acid	C_10_H_7_NO_3_	[M − H]^−^	188.0354
5	12.50	Linoleic acid	C_18_H_32_O_2_	[M − H]^−^	279.2329
6	7.57	Undecanoic acid	C_11_H_22_O_2_	[M − H]^−^	185.1547
7	1.99	Galangin	C_15_H_10_O_5_	[M + H]^+^	271.0597
8	2.04	5,7-Dihydroxychromone	C_9_H_6_O_4_	[M − H]^−^	177.0192
9	2.59	Sodium 4-hydroxy-benzoate	C_7_H_6_O_3_	[M − H]^−^	137.0246
10	1.47	Myricetin	C_21_H_20_O_12_	[M + H]^+^	465.1030
11	4.18	Demethylwedelolactone	C_15_H_8_O_7_	[M − H]^−^	299.0202
12	12.86	Gallic acid	C_7_H_6_O_5_	[M − H]^−^	169.0143
13	2.14	Citrate	C_6_H_8_O_7_	[M − H]^−^	191.0195
14	2.62	m-Xylene	C_8_H_10_	[M + H]^+^	107.0854
15	0.61	Nicotinamide	C_6_H_6_N_2_O	[M + H]^−^	123.0551
16	0.70	Catechol	C_6_H_6_O_2_	[M − H]^−^	109.0296
17	0.82	Methylgallate	C_8_H_8_O_5_	[M − H]^−^	183.0300
18	11.80	Oleic acid	C_18_H_34_O_2_	[M − H]^−^	281.2483
19	12.53	alpha-Linolenic acid	C_18_H_30_O_2_	[M + H]^+^	279.2318
20	0.56	Adenine	C_5_H_5_N_5_	[M + H]^+^	136.0618
21	3.35	Morin	C_15_H_10_O_7_	[M + H]^+^	303.0495
22	0.60	Nicotinic acid	C_6_H_5_NO_2_	[M + H]^+^	124.0392
23	4.20	Kaempferol	C_15_H_10_O_6_	[M + H]^+^	287.0546
24	0.92	Gentisic acid	C_7_H_6_O_4_	[M − H]^−^	153.0193
25	3.25	4-hydroxyphenylacetic acid	C_8_H_8_O_3_	[M − H]^−^	151.0402
26	0.03	Wogonin	C_16_H_12_O_5_	[M + H]^+^	285.0758
27	2.15	Isorhamnetin	C_16_H_12_O_7_	[M + H]^+^	317.0648
28	1.33	Ellagic Acid	C_14_H_6_O_8_	[M − H]^−^	300.9989
29	0.83	Caffeine	C_8_H_10_N_4_O_2_	[M + H]^+^	195.0875
30	1.22	7,8-Dihydroxycoumarin	C_9_H_6_O_4_	[M + H]^+^	179.0337
31	0.80	Kojic Acid	C_6_H_6_O_4_	[M + H]^+^	143.0340
32	2.70	cuminyl alcohol	C_10_H_14_O	[M + H − H_2_O]^−^	133.1012
33	1.62	vanillic acid	C_8_H_8_O_4_	[M − H]^−^	167.0351
34	4.58	3,4-Dimethoxybenzaldehyde	C_9_H_10_O_3_	[M + H]^+^	167.0702
35	3.36	Quercetin	C_15_H_10_O_7_	[M − H]^−^	301.0349
36	11.79	Fraxetin	C_10_H_8_O_5_	[M + H]^+^	209.0444
37	1.55	Deoxyvasicinone	C_11_H_10_N_2_O	[M + H]^+^	187.0865
38	11.86	Phthalic anhydride	C_8_H_4_O_3_	[M + H]^+^	149.0232
39	12.29	2-Hydroxy-4-methoxybenzaldehyde	C_8_H_8_O_3_	[M + H]^+^	153.0546
40	4.03	Zingerone	C_11_H_14_O_3_	[M − H]^−^	193.0872
41	1.38	p-Hydroxy-cinnamic acid	C_9_H_8_O_3_	[M − H]^−^	163.0402
42	0.85	Sesamol	C_7_H_6_O_3_	[M + H]^+^	139.0388
43	0.67	Bergenin		[M − H]^−^	327.0722
44	8.52	2-Phenylethyl formate	C_9_H_10_O_2_	[M + H]^+^	151.0753
45	7.92	Di-2-furanylmethane	C_9_H_8_O_2_	[M + H]^+^	149.0597
46	1.09	p-Mentha-1,3,8-triene	C_10_H_14_	[M + H]^+^	135.1167
47	5.21	Diethyl-phthalate	C_12_H_14_O_4_	[M − H]^−^	221.0821
48	1.74	Caffeic acid	C_9_H_8_O_4_	[M + H]^+^	181.0494
49	2.52	2′,4′-Dimethylacetophenone	C_10_H_12_O	[M + H]^+^	149.0961
50	13.70	Pyridoxine	C_8_H_11_NO_3_	[M + H]^+^	170.0811
51	4.14	Thymol	C_10_H_14_O	[M + H]^+^	151.1118
52	2.15	Quercetin	C_21_H_20_O_11_	[M − H]^−^	447.0933
53	9.84	4-tert-Butylphenol	C_10_H_14_O	[M + H]^+^	151.1118
54	1.75	2,4-Dimethylbenzaldehyde	C_9_H_10_O	[M + H]^+^	135.0805
55	0.35	5,7-dihydroxy-6-methoxy-2-phenylchromen-4-one	C_16_H_12_O_5_	[M + H]^+^	285.0757
56	1.97	P-Anisic acid	C_8_H_8_O_3_	[M + H]^+^	153.0547
57	8.36	2-Methoxybenzaldehyde	C_8_H_8_O_2_	[M + H]^+^	137.0598
58	4.31	4-Hydroxybenzoic acid	C_7_H_6_O_3_	[M + H]^+^	139.0388
59	4.05	Dihydrocapsaicin	C_18_H_29_NO_3_	[M + H]^+^	308.2220
60	1.59	Suberic acid	C_8_H_14_O_4_	[M − H]^−^	173.0819
61	5.25	2-Phenylethyl octanoate	C_16_H_24_O_2_	[M + H]^+^	249.1851
62	9.90	Arachidonic acid (not validated)	C_20_H_32_O_2_	[M + H]^+^	305.2472
63	3.38	Flavonol base + 4O, 1MeO	C_16_H_12_O_8_	[M − H]^−^	331.0461
64	2.22	Methylisoeugenol	C_11_H_14_O_2_	[M + H]^+^	179.1065
65	2.22	Methylisoeugenol	C_11_H_14_O_2_	[M + H]^+^	179.1065
66	1.02	alpha-Methylstyrene	C_9_H_10_	[M + H]^+^	119.0855
67	1.71	7-Methoxycoumarin	C_10_H_8_O_3_	[M + H]^+^	177.0545
68	11.09	9-Trans-Palmitelaidic acid	C_16_H_30_O_2_	[M − H]^−^	253.2173
69	2.86	Meperidine	C_15_H_21_NO_2_	[M + H]^+^	248.1642
70	1.91	4-Hydroxyphthalide	C_8_H_6_O_3_	[M + H]^+^	151.0389
71	5.81	Sinensetin	C_20_H_20_O_7_	[M + H]^+^	373.1286
72	0.86	Epicatechin	C_15_H_14_O_6_	[M − H]^−^	289.0718
73	5.56	1-(2-Furanyl)-1-propanone	C_7_H_8_O_2_	[M + H]^+^	125.0596
74	0.97	Piperonylic Acid	C_8_H_6_O_4_	[M − H]^−^	165.0194
75	0.95	Xanthoxyline	C_10_H_12_O_4_	[M + H]	197.0810
76	10.06	Dibutylphthalate	C_16_H_22_O_4_	[M + H]^+^	279.1585
77	3.84	Valerophenone	C_11_H_14_O	[M + H]^+^	163.1116
78	0.67	Monomethyl phthalate	C_9_H_8_O_4_	[M + H]^+^	181.0494
79	0.94	2,6-Dimethoxyphenol	C_8_H_10_O_3_	[M + H]^+^	155.0702
80	10.12	Acetophenone	C_8_H_8_O	[M + H]^+^	121.0647
81	1.52	Rubrofusarin	C_15_H_12_O_5_	[M + H]^+^	273.0754
82	13.70	Methyl 2-aminobenzoate	C_8_H_9_NO_2_	[M + H]^+^	152.0706
83	3.19	Loureirin A	C_17_H_18_O_4_	[M + H]^+^	287.1280
84	3.87	dihydrodamascenone	C_13_H_20_O	[M + H]^+^	193.1585
85	10.83	3-(4-Methoxyphenyl)-2-propen-1-ol	C_10_H_12_O_2_	[M + H]^+^	165.0911
86	4.69	Methyl linoleate	C_19_H_34_O_2_	[M + H]	295.2631
87	11.58	Phenylacetaldehyde	C_8_H_8_O	[M + H]^+^	121.0647
88	6.95	Atractylodin	C_13_H_10_O	[M + H]^+^	183.0806
89	1.17	Eugenin	C_11_H_10_O_4_	[M + H]^+^	207.0650
90	0.60	Formononetine	C_16_H_12_O_4_	[M − H]^−^	267.0724
91	5.84	Asarylaldehyde	C_10_H_12_O_4_	[M + H]^+^	197.0810
92	2.15	Dimethyl succinate	C_6_H_10_O_4_	[M + H]^+^	147.0650
93	0.52	1-Hexanethiol	C_6_H_14_S	[M + H]^+^	119.0896
94	0.71	Indole	C_8_H_7_N	[M + H]^+^	118.0649

**Table 2:** Differential metabolite summary.

No.	ESI	R_t_ (min)	Name of Metabolite	Molecular Formula	Molecular Weight	Measured Value	Ms/Ms	Trend
1	−	7.91	L-Palmitoyl Carnitine	C_23_H_45_NO_4_	399.3349	458.3469	M + CH_3_COO	↑
2	−	2.61	Quinolinic acid	C_7_H_5_NO_4_	167.0219	226.0357	M + CH_3_COO	↓
3	−	0.77	D-Mannose	C_6_H_1_2O_6_	180.0634	215.0333	M + Cl	↓
4	+	1.47	7-Methylxanthine nucleoside	C_11_H_15_N_4_O_6_ +	299.099	384.0649	M + K + HCOOH	↑
5	−	12.17	Ceramide	C_42_H_81_NO_3_	647.6216	706.6345	M + CH_3_COO	↓
6	−	5	D-Cysteine	C_3_H_7_NO_2_S	121.0197	241.0389	2M − H	↓
7	−	7.73	L-Tartaric acid	C_4_H_6_O_6_	150.0164	299.0261	2M − H	↓
8	−	1.57	L-(-)-3-Phenyl lactic acid	C_9_H_1_0O_3_	166.063	331.1139	2M − H	↑
9	−	3.31	3-Iodotyrosine	C_9_H_1_0INO_3_	306.9705	327.947	M + Na − 2H	↓
10	−	0.76	L-Glutamine	C_5_H_1_0N_2_O_3_	146.0691	145.062	M − H	↑
11	−	7.68	Puromycin	C_2_2H_2_9N_7_O_5_	471.223	523.2173	M + Cl + NH_3_	↓
12	−	1.15	O-Acetyl-L-serine	C_5_H_9_NO_4_	147.0532	146.0459	M − H	↑
13	−	5.68	L-Thyroxine	C_15_H_1_1I_4_NO_4_	776.6867	775.6784	M − H	↑
14	−	10.79	Neuronic acid	C_2_4H_4_6O_2_	366.3498	366.3452	M−	↑
15	−	8.35	α-Aminopropionitrile	C_3_H_6_N_2_	70.0531	91.0222	M + Na-2H	↓
16	−	10.06	9,12-Octadecatetraenoic acid	C_18_H_28_O_2_	276.2089	275.2016	M − H	↑
17	−	8.78	Trichloroethylene epoxide	C_2_HCl_3_O	145.9093	197.9061	M + Cl + NH_3_	↓
18	−	1.54	S-sulfo-L-cysteine	C_3_H_7_NO_5_S_2_	200.9766	259.9989	M + CH_3_COO	↑
19	−	3.04	Molybdate	H_2_MoO_4_	163.9007	326.7945	2M − H	↓
20	−	2.4	cis,cis-muconic acid	C_6_H_6_O_4_	142.0266	163.0926	M + Na − 2H	↓
21	−	3.05	trichloroacetic acid	C_2_HCl_3_O_2_	161.9042	160.8968	M − H	↓
22	−	8.6	1-Behenoyl-2-hydroxy-sn-glycero-3-phosphocholine	C_3_0H_6_2NO_7_P	579.4264	638.4395	M + CH_3_COO	↓
23	−	6.95	(20S)-Cholesta-5-ene-3beta,17,20-triol	C_27_H_4_6O_3_	418.3447	453.3114	M + Cl	↓
24	−	9.9	1,2-Dipalmitoyl-sn-glycero-3-phosphocholine	C_40_H_7_6NO_8_P	729.5309	748.5461	M + F	↓
25	−	6.97	Chlorophyll	C_33_H_32_MgN_4_O_3_	556.2325	555.2222	M − H	↓
26	−	12.15	Glucose ceramide (d18:1/16:0)	C_40_H_77_NO_8_	699.5649	758.5798	M + CH_3_COO	↑
27	−	4.48	Piperidine	C_5_H_11_N	85.0891	166.0692	M + Cl + HCOOH	↑
28	+	4.11	(E)-3-Hexen-1-ol	C_6_H_12_O	100.089	218.2116	2M + NH_4_	↑
29	+	5.9	Sterols	C_27_H_46_O	386.355	450.3783	M + CH_3_CN + Na	↓
30	+	6.54	Sphingomyelin	C_18_H_39_NO_2_	301.298	302.3059	M + H	↑
31	+	4.8	5β-hydrocortisone	C_21_H_30_O_5_	362.209	363.2164	M + H	↓
32	+	3.28	Dimethyl sulfoxide	C_2_H_6_O_2_S	94.009	141.018	M + HCOO + 2H	↓
33	+	0.86	Dimethyl sulfoxide	C_2_H_6_OS	78.014	79.0215	M + H	↑
34	+	2.05	Thymine	C_5_H_6_N_2_O_2_	126.043	127.05	M + H	↓
35	+	0.76	L-Gulose	C_6_H_1_2O_6_	180.063	383.1162	2M + Na	↓
36	+	3.28	Ascorbic acid	C_6_H_8_O_6_	176.032	353.0712	2M + H	↓
37	+	1.54	L-Ascorbic acid	C_14_H_17_NO_7_	311.101	312.111	M + H	↓
38	+	9.19	Osteotriol (Vitamin D3)	C_27_H_44_O_3_	416.329	417.3361	M + H	↓
39	+	1.5	3-Hydroxybutyric acid	C_4_H_8_O_3_	104.047	105.0539	M + H	↑
40	+	1.49	N-succinyl-LL-2,6-diaminopimelic acid	C_11_H_18_N_2_O_7_	290.111	337.1159	M + HCOO + 2H	↑
41	+	2.79	5-deoxy-5-methylthioadenosine	C_11_H_15_N_5_O_3_S	297.09	298.0969	M + H	↓
42	+	1.46	(2S,3S)-Butane-2,3-diol	C_4_H_10_O_2_	90.068	73.0647	M + H − H_2_O	↓
43	+	9.9	dihomo-γ-linolenic acid	C_20_H_34_O_2_	306.256	348.2906	M + CH_3_CN + H	↓
44	+	7.67	LPC(22:6/0:0)	C_30_H_50_NO_7_P	567.333	569.3326	M + H	↓
45	+	1.49	Ketodeoxynonanoic acid	C_9_H_16_O_9_	268.079	233.0672	M + H − 2H_2_O	↑
46	+	1.47	3,7-Dimethyluronic acid	C_7_H_8_N_4_O_3_	196.06	265.0532	M + Na + HCOOH	↑
47	+	0.77	Hydroxyacetone	C_3_H_6_O_2_	74.037	97.028	M + Na	↓
48	+	12.01	Oleate	C_18_H_34_O_2_	282.256	283.2638	M + H	↓
49	+	1.48	Nicotinamide mononucleotide	C_11_H_15_N_2_O_8_P	334.057	352.0822	M + NH_4_	↑
50	+	12.61	5-Hydroxy-2-oxo-4-ureido-2,5-dihydro-1H-imidazole-5-carboxylate	C_5_H_6_N_4_O_5_	202.034	316.9047	M − 2H + 3K	↑

Note: ESI +: positive ion mode; ESI −: Negative ion mode; (↑): up-regulated; (↓): down-regulated.

## Data Availability

The data that support the findings of this study are available from the corresponding author upon reasonable request.

## Supplementary Materials

The following supporting information can be downloaded at: https://www.mdpi.com/article/10.3390/molecules28217301/s1, Figure S1: TICof non-targeted metabolomics (A) positive ion mode; (B) negative ion mode.

## Sample Availability

Samples of the compounds are not available from the authors.

## Abbreviations

AJ: *Ardisia japonica* (Thunb) Blume; PK, peach kernel positive control; CON, control; MOD, model; WBV, whole blood viscosity; PV, plasma viscosity; HCT, hematocrit RBC; HSV, high shear viscosity; LSV, low shear viscosity; EDI, erythrocyte deformation index; ESR-K, the k-value of the erythrocyte sedimentation equation; EAI, erythrocyte aggregation index; LC-MS, liquid chromatography-mass spectrometry; TIC, total ion chromatograms; QC, quality control; VIP, variable importance prediction; AD, Alzheimer’s disease; HER, epidermal growth factor receptor; PI3K, phosphatidylinositol kinase-3; SAPK, stress-activated protein kinase; COX2, cyclooxygenase-2; Src, sarcoma gene; TNF, tumor necrosis factor; NF-κB, nuclear factor-kappa B complex; RIP, receptor-interacting protein; IKK, inhibitor of kappa B kinase; MAPK14, human mitogen activated protein kinase 14; PI3K-AKT, phosphatidylinositol 3 kinase(PI3K)/protein kinase B(AKT); H9c2, cardiomyocytes; Bcl-2, B-cell lymphoma-2; RBP4, retinol binding protein 4; TNF, tactical nuclear forces; IL-6, interleukin-6; COX-2, cyclooxygenase-2; iNOS, inducible nitric oxide synthase; JNK, c-Jun N-terminal kinase; ERK, extracellular regulated protein kinases; AKT1, rac-alpha serine/threonine-protein kinase; EGFR, epidermal growth factor receptor; HSP90AA1, heat shock protein HSP 90-α; PIK3CA, phosphatidylinositol-4, 5-bisphosphate 3-kinase catalytic subunit alpha isoform; TP53, recombinant tumor protein P53; VEGFA, vascular endothelial growth factor A; MMP9, recombinant matrix metalloproteinase 9; PLG, recombinant plasminogen; MMP2, matrix metalloproteinase 2; MAPK14, recombinant mitogen activated protein kinase 14; STAT1, recombinant signal transducer and activator of transcription 1; BP, biological processes; CC, cellular component; MF, molecular function; KEGG, Kyoto encyclopedia of genes and genomes; PDB, protein structure database; GO, gene ontology; QSBS, Qi stagnation and blood stasis; QDBS, Qi deficiency and blood stasis; ICAM-1, intercellular adhesion molecule-1.
