# Supplementary figures and images for: Investigating the Underlying Mechanisms of Ardisia japonica Extract’s Anti-Blood-Stasis Effect via Metabolomics and Network Pharmacology
Source: Molecules. 2023 Oct 27;28(21):7301. doi: 10.3390/molecules28217301 (PMC10649676; doi:10.3390/molecules28217301)

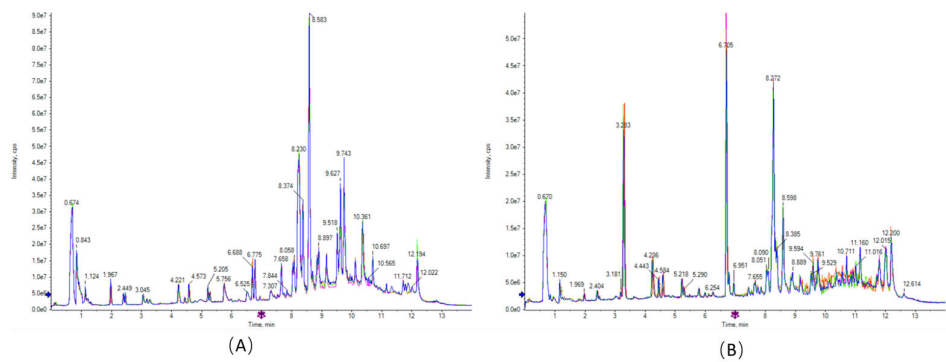

**Figure S1.** TICof non-targeted metabolomics (A) positive ion mode; (B) negative ion mode.

Supplement: Supplementary file 1 [file molecules-28-07301-s001.zip › Figure S1.pdf]
